# Intraocular injectable hydrogels for the delivery of cells and nanoparticles

**DOI:** 10.1016/j.mtbio.2025.101767

**Published:** 2025-04-12

**Authors:** Elena Ibeas Moreno, María José Alonso, Anna Abbadessa

**Affiliations:** a Center for Research in Molecular Medicine and Chronic Diseases (CiMUS), University of Santiago de Compostela, Campus Vida, 15782, Santiago de Compostela, Spain; b Department of Pharmacology, Pharmacy and Pharmaceutical Technology, School of Pharmacy, University of Santiago de Compostela, Campus Vida, 15782, Santiago de Compostela, Spain; c Health Research Institute of Santiago de Compostela (IDIS), University of Santiago de Compostela, Campus Vida, 15782, Santiago de Compostela, Spain; d Madrid Institute for Advanced Studies in Nanoscience (IMDEA), 28049, Madrid, Spain

**Keywords:** Hydrogel, Injectable, Eye, Nanoparticle, Cells, Ocular regeneration, Ocular drug delivery

## Abstract

The rising global life expectancy has led to a growing prevalence of ophthalmic diseases, while current treatments face important limitations in terms of efficacy, costs, and patient compliance. The use of injectable hydrogels as drug and cell carriers is a promising approach, compared to the injection of drug solutions or cell suspensions. This is because the hydrogel matrix may offer protection against clearance or degradation, may modulate drug/cell release, and provide a biomimetic substrate for differentiating cells while being minimally invasive. On one hand, injectable hydrogels for ocular drug delivery have been traditionally designed to host and release small drugs or proteins. However, limitations such as high burst release and difficulty of entrapping hydrophobic molecules led to the emergence of nanocomposite hydrogels, where the drug is entrapped in nanoparticles prior hydrogel incorporation. Composite systems offer great advantages over the injection of particle suspensions, improving particle fate and drug release kinetics. On the other hand, injectable hydrogels offer a cell-friendly environment to seek tissue regeneration, providing biomechanical and biochemical cues for cellular cross-talk, differentiation, and formation of new extracellular matrix. This review critically discusses recent advancements in the development of novel injectable hydrogels as delivery vehicles for drug-loaded nanoparticles and cells, with a major focus on the formulation components, administration routes, and other factors affecting performance, highlighting promising aspects and challenges to address in the future.

## Highlights

• Intraocular injectable hydrogels loaded with cells or drug-loaded particles are gaining a strong attention.
• Cell-laden hydrogels are promising for ocular tissue regeneration.
• Hydrogels hosting drug-loaded nanoparticles fine-tune drug release and biodistribution.
• The review discusses advantages and challenges of ocular hydrogels loaded with cells or nanoparticles.

## Graphical abstract

**Figure:**
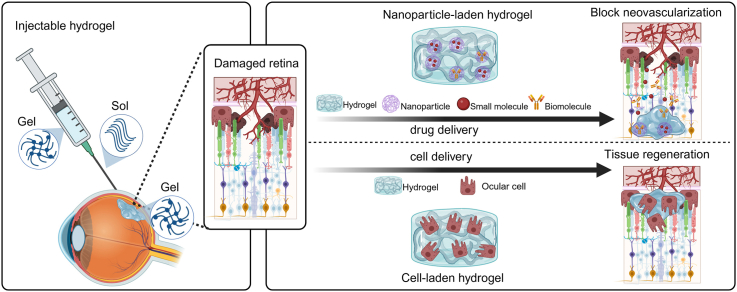

## 1 Introduction

According to the World Health Organization (WHO) at least 2.2 billion people suffer from vision impairment, with a huge economic global burden reaching 411 billion US$ in productivity loss [1]. As an example, patients with main eye dysfunctions will increase to 7.99 million by 2030, representing an increase of 103 % compared to 2021. By 2030, the accumulative cost of all pathologies is projected to reach 99.8 billion euros with the majority attributed to direct non-healthcare costs (44 %) and productivity losses (38 %). Direct healthcare costs represent the remaining 18 %. Glaucoma and diabetic macular edema will incur the highest individual costs, with 33.6 billion and 19.8 billion euros, respectively [2]. These numbers consistently rise due to the extended life expectancy and negative lifestyle changes, including sedentary behaviour and unhealthy dietary habits [3].

Current options for treating eye diseases mainly rely on surgery to remove or correct the damaged tissue or repetitive intraocular injections to deliver drugs *in situ*. Surgeries are limited to specific eye diseases, such as glaucoma, diabetic retinopathy (DR), retinal detachment, and cataracts, where these procedures can reduce intraocular pressure (IOP), replace the vitreous humor with a saline solution or a gas, correct the vision or replace damaged corneas, respectively. Nevertheless, in most of the cases, these surgical procedures aim to tackle symptoms, and do not offer a definitive solution to vision loss [4]. On the other hand, drugs are crucial for treating eye diseases, however, delivering them effectively, minimizing side effects and injection frequency remains a challenge. Ocular drug delivery is especially challenging in posterior segment's diseases affecting the retina or the optic nerve, such as age-related macular degeneration (AMD), DR, glaucoma, or intraocular tumors. In fact, unlike the anterior segment, the posterior segment of the eye is difficult to reach due to several physiological barriers that prevent the penetration of foreign substances. This leads to a great challenge in delivering active agents. Moreover, in the case of tissue-degenerating diseases, like AMD, most of the drugs act against the symptoms, *e.g.*, neovascularization, without tackling tissue degeneration which remains a critical unmet need for pharmacological treatments. This often leads to a mere delay in disease progression rather than a reversal or significant improvement in the underlying condition.

To overcome this challenge, several clinical trials are testing the potential of cell transplantation for tissue-degenerating eye diseases [5]. Cells can be transplanted as cellular suspensions, i.e., cells suspended in a medium, or cellular sheets, i.e., cells organized in a layer. However, both have limitations. Studies have shown that intraocular injection of retinal cell suspensions, including those containing Retinal Pigment Epithelium (RPE) or photoreceptor cells, often leads to poor cell survival and limited tissue regeneration [6,7]. This is likely due to the absence of a cell-instructive substrate. In fact, cells require signals from the surrounding extracellular matrix (ECM) and other cells to function properly and maintain their characteristic morphology and activity [8]. On the other hand, cell sheets offer the advantage of intercellular connections between differentiated cells, however, their implantation is technically challenging and invasive [[9], [10], [11]].

This highlights the need for new strategies to treat eye diseases in a minimally invasive and highly effective manner. In this context, injectable hydrogels stand out as promising biomaterials, as they are hydrophilic polymeric networks able to host and deliver cells and therapeutic agents [[12], [13], [14], [15], [16]]. Injectable hydrogels typically exist as liquids before or during the injection and jellify *in situ* after injection. This property may be due to a temperature-sensitive behaviour of the polymer network (thermogelation) [[17], [18], [19], [20]] or to the establishment of reversible interactions, such as ionic or dynamic covalent crosslinks, which can be broken during the injection and quickly reformed after deposition (shear-thinning, fast-recovering hydrogels) [[16], [17], [18]].

**Fig. 1:**
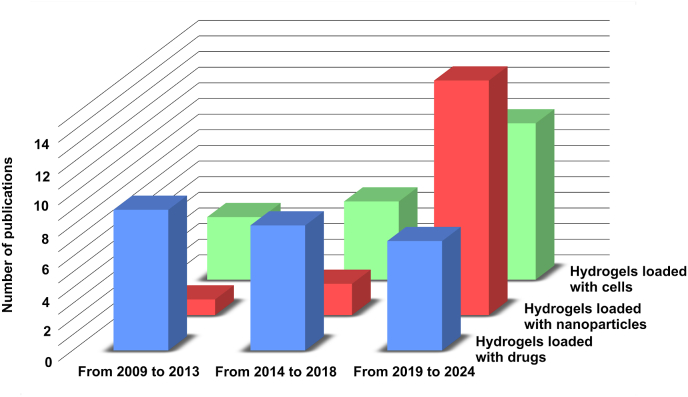
Publication trends of scientific papers related to the ocular delivery of hydrogels loaded with drugs, hydrogels loaded with NPs, and hydrogels loaded with cells. This figure was generated through a systematic literature review on Google Scholar, encompassing studies published between 2009 and 2024. Bibliographic data were categorized chronologically and processed using Excel to construct a graphical representation. Finally, image quality was improved using GIMP 2.1.34.

During the last 15 years, injectable hydrogels have been deeply investigated for the ocular delivery of (i) drugs (including small molecules and proteins), (ii) drug-loaded nanoparticles (NPs) and (iii) cells. The rationale behind the incorporation of such active agents into hydrogels lies on the fact that hydrogels may protect them from the stress generated during the injection or from *in vivo* clearance mechanisms, may contribute to the sustained release of drugs, and can offer an ECM-like substrate for cells [21,[22], [23], [24]]. As illustrated in Fig. 1, the incorporation of free drugs into hydrogels to seek sustained release is a strategy that has been more extensively explored in the past, as the majority of these papers have been published before 2018 and the number of publications related to this strategy is gradually decreasing overtime. In contrast, a new emerging approach involves the inclusion of drug-loaded NPs within hydrogel networks to yield nanocomposites. These are multiphase systems in which at least one possesses nanoscale dimensions [25]. Injections of drug-laden NPs in the eye offer promising advantages, such as the encapsulation of hydrophobic drugs. Nevertheless, when NPs are not trapped in a matrix, they present obstacles associated with toxicity due to initial burst release and low efficacy due to the rapid NPs clearance. Hydrogels overcome these limitations by modulating the release kinetics and providing protection against clearance mechanisms [[26], [27], [28]]. As shown in Fig. 1, most of the papers related to this strategy have been published in the last 6 years, highlighting the novelty of this approach.

Research on hydrogel-mediated delivery of cells to the eye has increased continuously over the last 16 years, notably during the last 6 years (Fig. 1). This is currently considered a promising approach for regenerating damaged ocular tissues as hydrogels provide a supportive and instructive environment for cells, ultimately leading to the formation of healthy and functional tissue [[29], [30], [31], [32]]. In some cases, researchers are also exploring the use of hydrogel-encapsulated cells as bioreactors, employing genetically-modified cells able to produce specific growth factors or antibodies, further promoting tissue repair [33,34,35].

In this review, we focused on hydrogels as intraocular delivery systems of cells and drug-loaded NPs, as these are the most novel and promising tendencies in the field. For the discussion on hydrogels solely containing free drugs we refer to previous reviews [[36], [37], [38]]. Promising aspects and challenges related to the formulation components, administration route, as well as fate of NPs and cells after injection are considered in depth. Hydrogel features are discussed based on the different polymers and crosslinking mechanisms used. Since a major focus is given on the formulation aspects and on how these affect the effectiveness of the delivery strategy, this review offers a guidance for the rational design of hydrogels for the ocular delivery of cells and drug-loaded NPs.

## 2 Anatomy of the eye

**Fig. 2:**
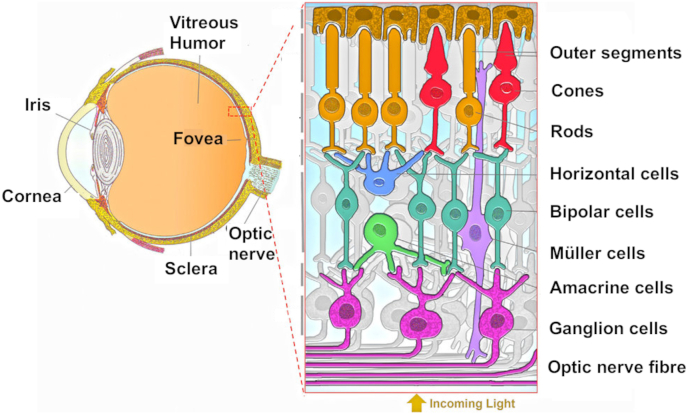
Illustration of the anatomy of the eye and retinal cells [39].

The eye is classified as one of the most complex organs of the body. Its major function is to capture the light coming from the outside and subsequently transform it into electrical signals, which are translated into visual images in the brain. Structurally, the eyeball is divided into two main regions, i.e., the anterior segment and the posterior segment, and their main structures are represented in Fig. 2.

The anterior segment comprises several structures. The cornea shields the eye from external irritants [40]; the iris governs the size of the pupil; the pupil is a circular hole, which allows light to pass through before reaching the lens [41]; the conjunctiva is involved in lubrication, immune defense and healing [42]; the ciliary body hosts the ciliary muscle, whose contraction results in the rounding of the lens [43]; the lens transmits and focuses light onto the retina [44]. The anterior chamber is filled with aqueous humor, which participates in transporting nutrients and immune cells, eliminating waste products, and facilitating light transmission [45].

The posterior segment includes several structures. The vitreous humor is a fluid comprising nearly 99 % of water and containing fibrillar proteins able to provide strength and flexibility [46]; the sclera maintains the necessary stiffness for the eye-wall [47]; the choroid is a vascular tissue supplying nutrients to the outer retina [48]. The retina serves as the conductor of light-to-neuronal electric signals, and it is composed of three cell types, namely photoreceptors, neuronal cells (horizontal, bipolar, amacrine and ganglion cells), and glial cells (Fig. 2) [[49], [50], [51]]. The optic nerve transports electric impulses generated by the retina to the visual area of the brain [52].

## 3 Eye diseases targeted by hydrogel-based therapies

Hydrogel-based therapies loaded with cells and nanoparticles are being investigated for various ocular diseases affecting the anterior and posterior segment. More than half of all ophthalmological diseases originate in the posterior segment of the eye. Left untreated, these conditions may cause irreversible vision loss [53]. Hence, the majority of the studies focus on four specific posterior segment diseases, which are the most prevalent worldwide, i.e., glaucoma, retinitis pigmentosa (RP), DR, and AMD [54].

### 3.1 Glaucoma

Glaucoma is a major cause of blindness due to the degeneration of the retinal ganglion cells and the optic nerve [55]. Its prevalence is expected to reach 112 million patients by 2040 [56]. This condition is characterized by a rise in the intraocular pressure resulting from an imbalance between the production and drainage of aqueous humor [57]. Traditional clinical approaches involve surgical procedures and topical medications [58]. Challenges of topical medications include their brief residence time before reaching the cornea, limited penetration, and low bioavailability within the eye. Therefore, if medications do not effectively control glaucoma, trabeculectomy as a surgical procedure is applied to drain out excess fluid [58]. Some patients experience complications after surgery, such as fibrosis cataracts, or even vision loss [59]. Due to all these limitations, recent attention has shifted towards advancing environmentally responsive drug delivery systems, particularly hydrogels [60].

### 3.2 Retinitis pigmentosa

RP is a genetic disorder involving the degeneration of rod photoreceptors, eventually leading to the degeneration of the RPE and blindness [61]. Efforts to enhance visual function by replacing damaged photoreceptor cells through cell transplantation have gained attention [[62], [63], [64]]. The main cell sources include retinal and stem cells [65]. However, in both strategies, cells suspended in a saline solution prove ineffective in neural regeneration. Instead, 3D matrices like hydrogels are preferable for their role in stimulating retinal regeneration [66].

### 3.3 Diabetic retinopathy

The number of people worldwide with diabetes is predicted to rise from 171 million in 2000 to 366 million in 2030. As the most common secondary effect of diabetes, DR is on a parallel upward trajectory [67]. The vascular endothelial growth factor (VEGF) is the growth factor playing the major role in this disease [68]. Treatments against DR encompass photocoagulation, intravitreal (IVT) injections of anti-VEGF agents and steroids, as well as vitreoretinal surgery [69]. However, monthly follow-up treatments pose challenges to patient adherence and incur high costs due to the repetitive practice [70]. The potential of an injectable hydrogel lies in enhancing delivery efficiency and enabling sustained release, which would reduce injection frequency [71].

### 3.4 Age-related macular degeneration

AMD is characterized by the loss of central vision, and it is expected to afflict 290 million people by 2040. In the wet form of AMD, choroidal neovascularization (CNV) occurs, leading to the rupture of the Bruch's membrane and abnormal invasion of new vessels in the subretinal space, contributing to the degeneration of the RPE and photoreceptors [72]. Current treatment involves monthly or bi-monthly IVT injections of anti-VEGF antibodies [73], which inhibit VEGF responsible for the formation of new blood vessels. However, this approach is invasive and repetitive, exhibits low patient compliance, incurs high costs, and does not lead to tissue regeneration. Considering these challenges, the implantation of healthy RPE cells is emerging as a promising clinical alternative. However, injecting cells suspended in a saline medium has shown low survival rates [74]. To overcome this challenge, cells require a 3D matrix that mimics ECM, making hydrogels an attractive clinical solution not only to prolong drug release kinetics but also to improve the survival of embedded cells [75].

## 4 Injectable hydrogels as delivery vehicles of cells and drug-loaded nanoparticles

### 4.1 Design principle and material selection for hydrogels loaded with cells and nano-encapsulated drugs

Hydrogels are promising delivery systems for cells and drug-loaded NPs targeting ocular diseases. Injectable hydrogels are crucial in biomedical engineering due to their ability to deliver drugs precisely in space and time under low pressure, using a minimally invasive approach through small needles or catheters compared to traditional hydrogels with higher viscosity [[76], [77], [78], [79]]. Traditional hydrogel materials usually display rigidity due to their irreversible chemistry which does not allow injection after cross-linking and may cause damage in the targeted tissue defect [80]. In contrast, injectable hydrogels offer a self-healing-like behaviour with the ability to adapt to the target anatomical tissue defect. The combined properties of injectable hydrogels make them highly promising for various biomedical applications, including tissue repair, controlled drug release, and cell protection [[81], [82], [83]].

**Fig. 3:**
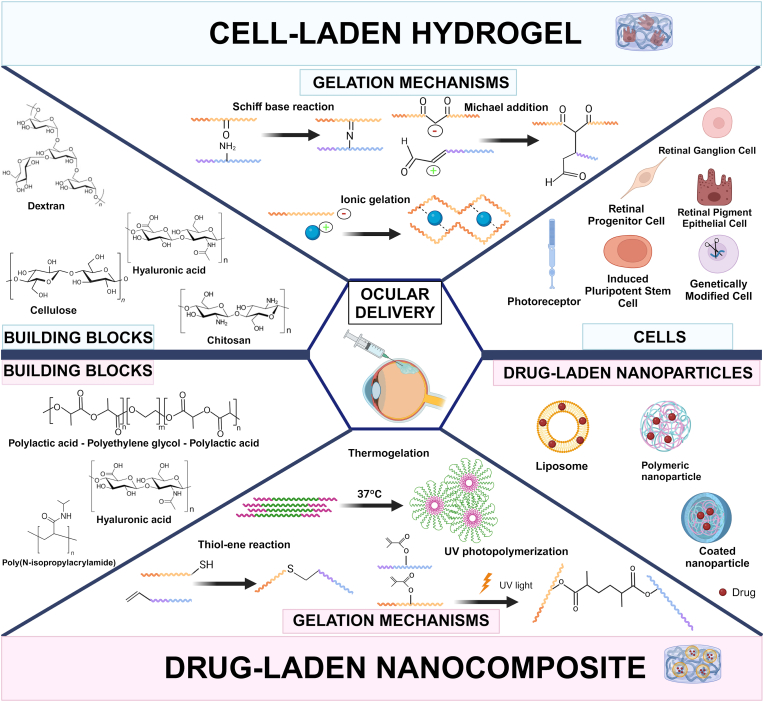
Schematic overview of the most common hydrogel components and crosslinking strategies employed in cell-laden hydrogels (upper panel) and drug-loaded nanocomposites (lower panel) for ocular delivery. Illustration created with https://BioRender.com.

As discussed in detail in the next sections, a clear distinction in the class of building blocks, hydrogel composition, crosslinking mechanisms and release kinetics arises depending on whether a cell-laden hydrogel or a NP-laden hydrogel is designed. Building blocks used for the cell-laden hydrogels are usually natural polymers, mainly polysaccharides, to simulate ECM, important for cell differentiation [[21], [22], [23], [24], [33], [74],^34^,[84], [85], [86], [87], [88], [89]], whereas those used for nanoparticle-loaded hydrogels are usually synthetic polymers to enable a more precise control over physico-chemical characteristics and drug/nanoparticle release kinetics [19,20,[90], [91], [92], [93], [94], [95], [96], [97]] (Fig. 3). Moreover, cell-laden hydrogels are often enriched with biomimetic cues for cell attachment, proliferation and differentiation [22,23,86], whereas nanoparticle-loaded hydrogels lack these components. Furthermore, crosslinking mechanisms used for cell-laden hydrogels are more often based on chemical reactions, which ensure long-term stability of the hydrogel, necessary for long-term cell differentiation [74,[22], [23], [24], [33],^34^,^75^,^86^,^89^,^98^], whereas hydrogels loaded with nanoparticles are generally crosslinked via physical gelation, mainly thermogelation [19,20,90,[92], [93], [94], [95],^97^].These differences in hydrogel composition and crosslinking also affect the different release profile of the included components (cells or nanoparticles). Chemically crosslinked hydrogels loaded with cells enable a slow-release mechanism and mainly act as a preliminary support until cells are able to produce their own ECM. In contrast, weaker hydrogels often used for NP-loaded systems may enable a faster release of the nanoparticles which will potentially migrate to the targeted tissue [99].

### 4.2 Crosslinking mechanisms

In the reviewed articles, the most common crosslinking mechanisms based on chemical reactions are those relying on Schiff base formation, Michael addition, thiol-ene chemistry and ultraviolet (UV)-polymerization [21,[22], [23], [24], [25],^35^,^78^,^92^,^94^,^99^,^100^,[101], [102], [103], [104]]. The Schiff base reaction establishes a dynamic covalent bond between aldehydes/ketones and amines/hydrazides [102,103]. This reaction occurs in aqueous solutions under physiological conditions, resulting in non-toxic products, and involving the nucleophilic attack of nitrogen on the carbonyl carbon [104,105]. Michael addition is a reaction where a Michael donor (a nucleophile, e.g., thiol or amine) reacts with a α,β-unsaturated carbonyl compound (e.g., acrylates or maleimides) acting as a Michael acceptor. It enables the formation of hydrogels under mild conditions, making it ideal for biocompatible and injectable hydrogels [106]. Thiol-ene chemistry is a click reaction between a thiol and an alkene under radical initiation to form a thioether, typically under UV light or thermal activation. This reaction is rapid, highly efficient, and oxygen-tolerant, making it useful for precise hydrogel crosslinking with tunable properties [107,108]. UV Polymerization is a photopolymerization process where vinyl moieties (e.g., acrylates) polymerize in response to a radical generation under the action of a photo-initiator and UV light exposure [109].

The most common physical crosslinking mechanisms exploited in the reviewed articles are thermogelation and ionic gelation [19,20,90,[92], [93], [94], [95],^97^]. Thermogelation is the reversible and temperature-dependent sol-gel transition of certain polymers in aqueous solutions, which depends on the Lower Critical Solution Temperature (LCST) of the polymer. Below the LCST, polymer chains remain hydrated and dissolved in water due to polymer-water interactions, whereas above the LCST, polymer-polymer interactions prevail, triggering phase separation and gel formation [110]. Many thermogelling polymers self-assemble into interconnected flower-like micelles [111] (Fig. 3). Finally, the ionic gelation is a cross-linking mechanism where polyanionic biopolymers interact with divalent cations. The most common example is that of alginate in which crosslinking occurs through the ‘egg-box’ structure formation, involving cooperative binding of divalent cations (i.e., Ca^2+^) to guluronic acid blocks, leading to network formation [112,113].

## 5 Injectable hydrogels loaded with cells

### 5.1 Cells

Tissue engineering has emerged as a promising strategy to repair tissue damage and restore its functions, by combining scaffolds, such as hydrogels, healthy cells, and bioactive molecules [114]. In the ocular field, the use of cell-laden hydrogels is currently under investigation to tackle degenerative ocular diseases. As reported in Table 1, this approach has been mainly applied to degenerative diseases affecting the retina, such as AMD, and in a few cases, also to diseases of the anterior segment of the eye, such as ocular surface damage after chemical burns or limbal stem cells (LSCs) loss.

**Table 1:** Hydrogel loaded with cells for ocular delivery. Description of the hydrogel building blocks, crosslinking mechanism (“*Chem*” and “*Phys*” indicate chemical and physical crosslinking, respectively), cell type, targeted disease and animal model.

Hydrogel building blocks	Crosslinking mechanism	Cell type	Disease and Animal Model	Ref.
CMHA-GeL-GSSG CMHA-Gel-GSSG-RGD	Michael addition *(Chem)*	ADSCs	Anterior segment eye diseases Rabbit model	Zarembinski et al., 2014 [22]
GeLSH-HAMA GeLSH-HAMA-PDA	Michael addition *(Chem)*	RPCs	Retinal degenerative diseases Mouse model	Tang et al., 2019 [23]
CMHA-GeL	Michael addition *(Chem)*	RPCs	Retinal degenerative diseases Mouse model	Liu et al., 2013 [24]
CS-oxDex	Schiff-Base *(Chem)*	RPCs	Retinal degenerative diseases Mouse model	Jiang et al., 2018 [74]
Ch-oxPEG Alg-GeL	Schiff base *(Chem)*	CAR-T cells	Retinoblastoma Mouse model	Wang et al., 2020 [33]
Laminin-CMC-oxDex	Schiff base *(Chem)*	CECs	Age-related macular degeneration Rat model	G.Pandala et al., 2025 [86]
CoL type I–HA-4SPEG	Amidation *(Chem)*	Genetically modified ARPE-19 (secreting sVEGFR)	Neovascular retinal diseases –	Konturii et al., 2014 [34]
GeLMa	UV-polymerization *(Chem)*	CjSCs	Conjunctival disorders –	Zhong et al., 2020 [98]
GeLMa GCMSs	UV -polymerization *(Chem)*	ARPE-19	Age-related macular degeneration Rat model	Cheng et al., 2023 [75]
GeL-HPA/HA-Tyr	Enzyme (HRP)-mediated *(Chem-IPN)*	hRGC	Glaucoma Rat model	Dromel et al., 2021 [89]
CoL type I-PNIPAAm	thermogelation *(Phys)*	RPE cells	Retinal degenerative diseases –	Fitzpatrick et al., 2010 [115]
CHC	thermogelation *(Phys)*	iPSCs	Surgical abrasion-injured corneas Rat model	Chien et al., 2012 [88]
HA-MC	thermogelation (*Phys*)	RPCs	Retinal degenerative diseases Mouse model	Ballios et al., 2010 [84]
HA-MC	thermogelation (*Phys*)	RSC-derived rods	Retinal degenerative disease Mouse model	Ballios et al., 2015 [21]
GG-HA	Ionic gelation *(Phys)*	ARPE-19	Age-related macular degeneration –	Youn et al., 2022 [87]
AP-CAC	Ionic gelation (*Phys*)	Genetically modified HEK293 (secreting GDNF)	Retinal degenerative diseases –	Yin Wong et al., 2022 [35]
GG-Ca^+2^	Ionic gelation *(Phys)*	MSCs	Corneal damage Rabbit model	Liu et al., 2024 [100]

ADSCs, adipose stem cells; Alg, alginate; AP, Alginate-poly-L-lysine; ARPE-19, a retinal pigment epithelium cell line; CAC, Composite alginate collagen; CAR-T cells; chimeric antigen receptor T cells; CECs, choroidal endothelial cells; CHC, carboxymethyl-hexanoyl chitosan; CjSCs, conjunctival stem cells; CMC, carboxymethyl-chitosan; CoL, collagen; CMHA, carboxymethyl hyaluronic acid; CS, chitosan hydrochloride; GCMSs, gelatin methacrylic chitosan microspheres; GDNF; glial cell-derived neurotrophic factor; GeL, gelatin; GeLMa; gelatin mathacrylamide; GeLSH, thiolated gelatin; GG, gellan gum; GSSG, oxidized gluthatione; HA, hyaluronic acid; HAMA, methacrylated hyaluronic acid; HEK293, human embryonic kidney 293 cell line; HPA, hydroxypheyl propionic acid; hRGCs; human retinal ganglion cells; HRP, horseradish peroxidase; IPN, interpenetrating network; iPSCs, induced pluripotent stem cells; MC, methylcellulose; MSCs, mesenchymal stem cells; oxDex, oxidized dextran; oxPEG, oxidized poly(ethylene glycol); PNIPAAm, poly(N-isopropylacrylamide); RGD, arginylglycylaspartic acid; RPCs, retinal progenitor cells; RPE, retinal pigment epithelium; RSC, retinal stem cells; Tyr, tyrosine; PDA, polydopamine; 4SPEG, poly(ethylene glycol) ether tetrasuccinimidyl glutarate; sVEGFR, secrete vascular endothelial growth receptor.

The character “-” in the animal model column indicates that no animal model has been reported.

#### 5.1.1 Types of cells incorporated into hydrogels

Differentiated cells such as ARPE-19, a RPE cell line, and primary RPE cells [75,87,115], as well as undifferentiated cells like retinal progenitor cells (RPCs) [74,23,24], mesenchymal stem cells (MSCs) as adipose-derived stem cells (ADSCs) [22] or induced pluripotent stem cells (iPSCs) [88] are currently under investigation for the development of injectable hydrogels against degenerative ocular diseases, including but not limited to AMD, DR, and RP. Among the undifferentiated cell types, RPCs are the predominant ones used in preclinical studies for encapsulation into injectable hydrogels. In contrast to mature mammalian retinal cells, which lack regenerative and differentiation capacity [116], RPCs, isolated from embryonic retinas, have demonstrated the ability to differentiate into diverse retinal cell types, including bipolar neurons, rod photoreceptors, and Müller glial cells [117]. Moreover, a Food and Drug Administration (FDA)-approved Phase I/II clinical trial (NCT02464436) based on the subretinal injection of human retinal progenitor cells (hRPCs), targeting RP, is ongoing to evaluate their safety, tolerability, and efficacy [118]. RPCs encapsulation in injectable hydrogels, employs cell densities ranging from 2∗10^3^ to 1∗10^6^ cells/mL for *in vitro* studies, and from 4∗10^4^ to 1∗10^7^ cells/mL, for *in vivo* studies. For example, Tang et al. developed a novel RPCs-laden hydrogel composed of thiolated gelatin (GelSH), methacrylated hyaluronic acid (HAMA), and the mussel-inspired adhesive molecule polydopamine (PDA) [23]. *In vitro* studies revealed that this hydrogel system supported high cell viability, cell protection from the injection mechanical stress, and enhanced cell adhesion mediated by the upregulation of Cadherin 4. Most notably, RPCs underwent retinal neuronal differentiation, as evidenced by the increased expression of Recoverin (a photoreceptor marker), β3-tubulin, and protein kinase C-α (Pkc-α) (a bipolar cell marker). *In vivo* experiments involving subretinal injection demonstrated the successful implantation and integration of RPCs in the retina [23]. Despite these promising aspects, RPCs face challenges in their translation to the clinic. Their use raises ethical concerns as retinas are isolated from human embryos between weeks 14th and 20th of gestation. Additional disadvantages involve their low proliferative capacity [119], and the need of differentiation and characterization steps before use.

To overcome this challenge, differentiated cells may be preferred in some cases. For example, adult RPE cells and the ARPE-19 cell line are the most commonly employed differentiated cells against AMD, where the RPE undergo degeneration [34,75,87,115]. Of these two cell types, ARPE-19 are more often encapsulated into injectable hydrogels compared to RPE cells (Table 1). In fact, only one study, conducted by Fitzpatrick et al., reported the successful encapsulation of RPE cells into a thermoresponsive hydrogel composed of poly(*N*-isopropylacrylamide) (PNIPAAm) and collagen type I (CoL I) [115]. This study highlights the crucial role of CoL I in promoting RPE cell attachment. Interestingly, it demonstrates that a pre-incubation period of RPE cells with the CoL-containing hydrogel precursor significantly enhanced cell retention within the constructs, compared to hydrogels that were rapidly formed after cell mixing. This is explained by the fact that in the latter case, cells did not have sufficient time to interact with CoL and were expelled during the temperature-induced phase separation of the hydrogel. The more common use of ARPE-19 over RPE cells is likely explained by the fact that ARPE-19 cells are a more robust cell type and are easily available in large quantities, compared to primary RPE cells which have to be isolated from human or animal tissues. Notably, ARPE-19 cells, included in injectable hydrogels, have been genetically modified to secrete vascular endothelial growth factor receptor 1 (sVEGFR1), with an active function in capturing and neutralizing VEGF, a key player in retinal neovascularization [34]. Genetic engineering is emerging as a promising approach to address neovascular retinal diseases by providing sustained drug delivery and regenerative capacity. Nevertheless, challenges exist in controlling expression levels and accurately assessing therapeutic efficacy [34]. It must be noted that ARPE-19 cells often present contamination from other cell types, derived from the cell line manipulation [75,87]. Although from this perspective RPE cells might be considered more promising [120], a clinical trial involving the transplantation of adult human allogeneic RPE cells resulted in unsatisfactory outcomes, with patients experiencing rejection, and grafts developing fibrosis after suspension of immunosuppression treatment [121].

To overcome graft rejection, MSCs have been proposed as a valid cell type for transplantation, as they exhibit immunomodulatory, anti-apoptotic, and anti-inflammatory effects, and are easily isolated from adipose tissue or the bone marrow (BM) [122]. However, to the best of our knowledge, only the study by Zarembinski et al. explored the use of ADSCs in injectable hydrogels for treating anterior segment diseases [22]. ADSCs specifically aim to address corneal defects, which in severe cases can result in LSCs deficiency. *In vivo* studies involving intracutaneous and subconjunctival injections have demonstrated biocompatibility over a two-week post-injection period [22]. Concerning the clinical potential of MSCs in the ocular field, a pilot clinical study conducted by Park et al. involved the injection of autologous CD34^+^ BM-MSCs into the vitreous cavity [123]. Preliminary findings suggest that the autologous cells were well tolerated, but their effectiveness in retinal regeneration remains unclear [123].

Interestingly, there is a notable disparity between preclinical and clinical strategies in selecting cellular sources for treating degenerative ocular diseases. While preclinical studies exploiting cells-laden injectable hydrogels prefer RPCs or RPE cells, clinical studies involving cells suspended in saline are predominantly focused on human embryonic stem cells (hESCs) and their potential differentiation into RPE cells. For instance, two clinical trials, NCT0134493 and NCT01345006, each involving 13 participants, evaluated the safety and tolerability of subretinal injections of hESCs-derived RPE cells, targeting Stargardt's macular dystrophy and dry AMD, respectively [120]. Following a four-month period, no signs of hyperproliferation, tumorgenicity, ectopic tissue formation, or rejection were observed in either study. Additionally, patients with Stargardt's macular dystrophy demonstrated an increase of pigmentation at the RPE site of transplantation, explained by the proliferation of RPE cells. However, this increase in pigmentation was not related to a significant improvement in visual function, including visual acuity or retinal sensitivity [120]. Generally speaking, embryonic stem cells (ESCs) offer a potentially unlimited source with easier procurement compared to RPCs [124]. Nevertheless, challenges related with the use of ESCs include a high proliferative capacity linked to the risk of tumor and teratoma formation, complexities in directing multidirectional differentiation, and ethical considerations that require careful evaluation in therapeutic applications [125]. As a matter of fact, to the best of our knowledge, no preclinical studies are exploring hESC in injectable hydrogels for ocular delivery.

Since 2006, when Takahashi and Yamanaka introduced the factors Oct3/4, Sox2, c-Myc, and Klf4, into mouse adult fibroblasts to induce the pluripotent stem state [126], induced pluripotent stem cells (iPSCs) technology has captured the scientific spotlight. Therefore, iPSCs are proposed to replace ESC-based therapies in retinal regeneration since they represent an unlimited cell source with low immunogenicity. iPSCs have also emerged as an alternative to limbal stem cells (LSCs) transplantation [127], for which clinical data demonstrated persistent inflammation, triggered by the amniotic membrane on which the cells are cultured [128]. To date, only one study, conducted by Chien et al., has explored the use of iPSCs in injectable hydrogels for ocular applications [88]. This study employed a novel approach by reprogramming human corneal keratocytes into iPSCs and encapsulating them in an injectable hydrogel, eliminating the need for amniotic membranes. Their findings demonstrated that the iPSCs loaded within a hydrogel significantly enhanced corneal repair in a model of induced corneal injury and LSCs deficiency [88]. This breakthrough approach holds the potential to revolutionize corneal regeneration therapy for patients with severe LSCs dysfunction and corneal defects [88]. Nonetheless, there is no currently FDA-approved iPSCs-based retinal treatment, due to its inherent high risk of gene mutation [129].

Another emerging approach involves the use of genetic engineering to obtain a *de novo* and sustained delivery of therapeutically active proteins using cells as bioreactors [34,35]. In this context, the chimeric antigen receptor T (CAR-T) cell therapies has been a breakthrough therapy, in the field of cancer. Clinical trials have already begun in lung cancer, ovarian cancer and breast cancer, among others [130]. On the preclinical front, CAR-T cells have reached retinoblastoma, promoting the eradication of tumor cells while preserving vision [33]. When encapsulated in a chitosan-oxidated polyethylene glycol (Ch-oxPEG) and alginate-gelatin (Alg-Gel) hydrogels, and injected into the subretinal space of mice, modified CAR-T cells showed promising results in reducing tumor size. However, some challenges still remain that need to be addressed before CAR-T cell therapy can be widely used to treat solid tumors, such as the heterogeneity of tumor antigens, the infiltration into tumor tissues, and the immunosuppressive tumor microenvironment [33].

In the context of genetic engineering, Yin-Wong et al., modified Human Embryonic Kidney (HEK) 293 cells to stimulate the secretion of glial cell-derived neurotrophic factor (GDNF) [35]. Notably HEK293 cells were used as drug-secreting cells to improve retinal structure, reduce photoreceptor apoptosis, and enhance adequate electrophysiological functions [25]. Building upon and going beyond existing research, in our opinion, an ambitious step forward in this direction will be the use of genetically engineered cells which simultaneously act as drug-secreting systems and as cell source for tissue regeneration. In this sense, a cell-laden hydrogel would have a dual function: remodeling and regenerating damaged tissue with healthy cells while allowing the co-delivery of therapeutic agents.

#### 5.1.2 Modalities of injection of hydrogels

**Fig. 4:**
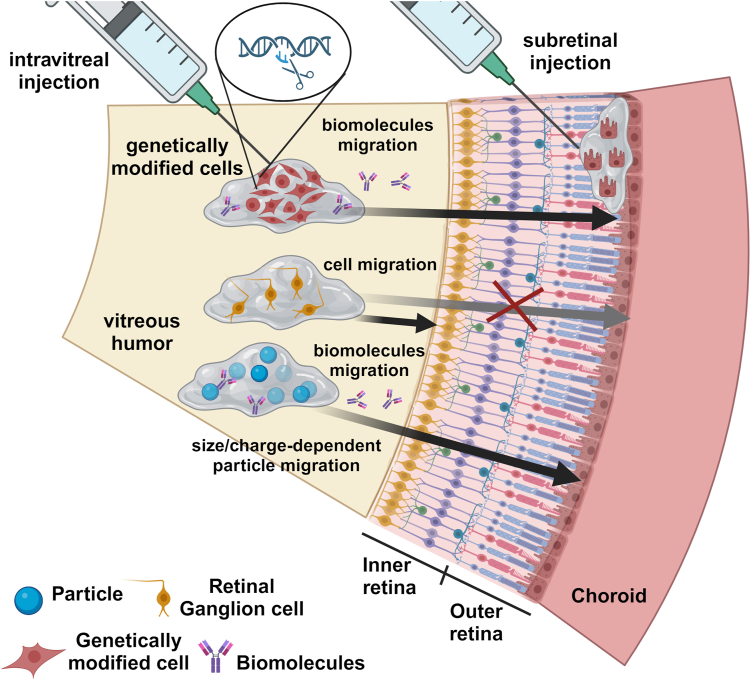
Diagram illustrating the IVT injection and subretinal injection of hydrogels loaded with cells or NPs. The less invasive IVT injection is preferred when the hydrogel is loaded with drug-secreting cells, cells targeting the inner retina or NPs. The subretinal injection is the preferred way to deliver cells in the subretinal space. Biomolecules released from genetically modified cells and particles are more likely to migrate through the retina, whereas particle migration strongly depends on particle size and surface charge. Illustration created with https://BioRender.com.

The administration route selected for a hydrogel containing cells, designed to support tissue regeneration, is determined by the location of the targeted tissue. In instances where the anterior eye segment is affected, periocular administration via subconjunctival injection onto the sclera is employed. In conjunctival disorders, conjunctival stem cells (CjSCs) are injected through the subconjunctival route to regenerate the conjunctiva [98,131]. In contrast, subretinal injection is the most widely employed technique for cellular delivery aimed at treating retinal degenerative diseases like AMD, DR, and RP [21,33,23,24]. In these cases, where the damaged cells (e.g., RPE cells or photoreceptors) are located in the outer retina, subretinal injection is preferred over the less invasive IVT injection (Fig. 4). This is because an IVT injection would result in low cellular viability and dedifferentiation due to diminished tissue specific cellular signals within the vitreous, as well as a tremendous difficulty for the cells to reach the outer retina due to the presence of several cellular layers (inner retina) located between the vitreous and the outer retina [132]. In line with this, only one study reported the IVT injection of cells for retinal regeneration [89]. In this study, human retinal ganglion cells (hRGCs) were successfully engrafted in the retinal ganglion cell (RGC) layer of the retina after IVT administration of cell-laden hydrogels against glaucoma [107]. We explain the success of this IVT cell delivery system by the fact that in the case of glaucoma, the targeted cellular layer, i.e. the RGC layer, is the retinal stratum nearest to the vitreous. Although these findings are encouraging in the glaucoma field, we envision that for degenerative diseases of the outer retina, the subretinal administration, which strategically positions cells between RPE and photoreceptors (Fig. 4), will remain the most efficient administration route. In this context, the sole exception arises when cells are employed as drug-secreting vehicles rather than being transplanted for tissue regeneration purposes. This is the case of the study by Yin-Wong et al. where genetically modified HEK 93 cells loaded in a hydrogel were intravitreally administered to deliver GDNF [35]. In this case, the reduction in photoreceptor apoptosis and retinal function loss is attributed to the diffusion of the delivered GDNF capable of reaching the outer retina [35].

#### 5.1.3 Effects of injection processes on cells

The choice of cellular density in injectable hydrogels for ocular applications is contingent upon the specific nature of the study. Generally speaking, the cell density used in the *in vitro* studies ranged from 2∗10^3^ to 5∗10^7^, whereas the cell density used in *in vivo* studies from 1∗10^3^ to 1∗10^9^. In most of the cases, the cellular density used in *in vivo* experiments is higher than that used in the corresponding *in vitro* study. This can be explained by the need to compensate for possible cell death caused by the *in vivo* cell injection. In fact, in *in vivo* studies, cells embedded into the hydrogel undergo the mechanical stress of an injection, passing through narrow needles with gauges ranging from 26 G to 30 G. Conversely, in *in vitro* studies, cells are gently placed in the hydrogel using a pipette, avoiding injection-related stress, and are maintained in a controlled environment with frequent medium changes for continuous nutrient renewal, ultimately improving cellular viability parameters. It is generally recognized that hydrogels may protect cells from the mechanical stress of the injection compared to cells in saline medium. However, this may depend on several factors including type of cells and hydrogels. For this reason, in our opinion, it is important to evaluate *in vitro* cell viability and behaviour, after injection through a narrow needle, prior *in vivo* experiments. Jiang et al. and Tang et al. injected RPCs embedded in a self-healing hydrogel using a 26G needle and showed a RPCs viability of 90 % and 95 % respectively, 3 h after injection [74,23]. Moreover, the morphology of the RPCs remained unchanged, demonstrating the hydrogel ability to protect cells from the mechanical stress during the injection. Additionally, Tang et al. evaluated the expression of proinflammatory cytokines, such as IL-6, and apoptotic factors, such as Caspase-3, finding significantly reduced expression levels in cells on hydrogel substrates compared to cells in medium [23]. Similarly, Cheng et al. encapsulated ARPE-19 cells in an injectable hydrogel and examined cell viability and reactive oxygen species (ROS) production as indicators of cell damage, after a 20 G injection [75]. The results indicated 80 % cell viability and low ROS production two days and four days after injection with no significant differences between these timepoints, further supporting the hydrogel protective effects on cells. Nevertheless, it has to be noted that the large-diameter needle (20 G) employed by Cheng et al. in their post-injection viability assay could lead to an underestimation of cell damage compared to *in vivo* injections with finer needles (e.g., 27 G), in which cells likely suffer more mechanical stress [75].

Among all the reviewed studies, only one conducted a cell density screening to identify the optimal cellular density for the aimed effect [34]. In this study, Konturi et al. tested cell densities between 5 and 80 million cells/ml to maximize the cellular secretion of sVEGFR1, and found an optimal value between 20 and 40 million cells/ml, with no significant differences in secretion ratio [34]. Beyond this range, cellular densities may lead to cell death as nutrient supply and waste removal are impeded.

### 5.2 Intraocular injectable hydrogels for cell delivery

Hydrogels have become an efficient delivery vehicle to transport cells to the front and the back of the eye. Naked transplanted cells without their native ECM, suffer rapid death. As complex structures, they require a substrate that mimics their ECM, providing specific survival cell signals [133]. Hydrogels, as water-rich networks, can improve cell viability and localization post-injection, avoiding anoikis, i.e., the programmed cell death after cell detachment from the ECM [134].

#### 5.2.1 Building blocks of hydrogels

In most of the studies, the building blocks used to fabricate cell-laden injectable hydrogels for ocular delivery are natural biopolymers, such as polysaccharides and proteins. Among polysaccharides, common examples include hyaluronic acid (HA), dextran (Dex), chitosan (Ch), gellan gum (GG), alginate (Alg), and cellulose [74,21,84,[87], [88], [89]]. These are used in their native form or are chemically modified with reactive groups to allow chemical cross-linking. On the other hand, common proteins used as building blocks are gelatin (GeL) and CoL I [33,24,89]. A limited number of studies involve hybrid hydrogels that combine natural and synthetic polymers. These incorporate synthetic components like PEG, poly(ethylene glycol) ether tetrasuccinimidyl glutarate (4SPEG), and PNIPAAm [33,34,115]. Among the studies on hybrid hydrogels, only the one reported by Wang et al. includes *in vivo* testing, highlighting the limited development of these systems compared to those solely made of natural polymers [33]. The rationale behind using natural polymers lies on the need to provide biomimetic cues for cell anchoring, cell proliferation, and in some cases cell differentiation. For example, Ch is known to activate some intracellular pathways responsible for cell proliferation, namely the phosphatidylinositol-3-kinase (PI3K)/protein kinase B (AKT) pathway and the mitogen-activated protein kinase (MAPK)/extracellular signal-regulated kinase (ERK) pathway [135,136]. In line, Jiang et al. showcased the proliferative effect on RPCs loaded in a hydrogel based on chitosan hydrochloride (CS) and oxidized dextran (CS-oxDex) [74]. Another example involves the triaminoacidic sequence arginine-glycine-aspartate (RGD). RGD is present in proteins such as CoL and GeL, and enhances cell attachment by interacting with transmembrane integrins [137]. An additional example of biopolymer/cell interaction is that between HA and the CD44 cell surface adhesion receptor. This interaction promotes the activation of cell proliferation and survival pathways [24]. The role of CD44 in cell survival has been investigated by Ballios et al. using an hyaluronic acid/methylcellulose (HAMC)-based injectable hydrogel [21].

**Fig. 5:**
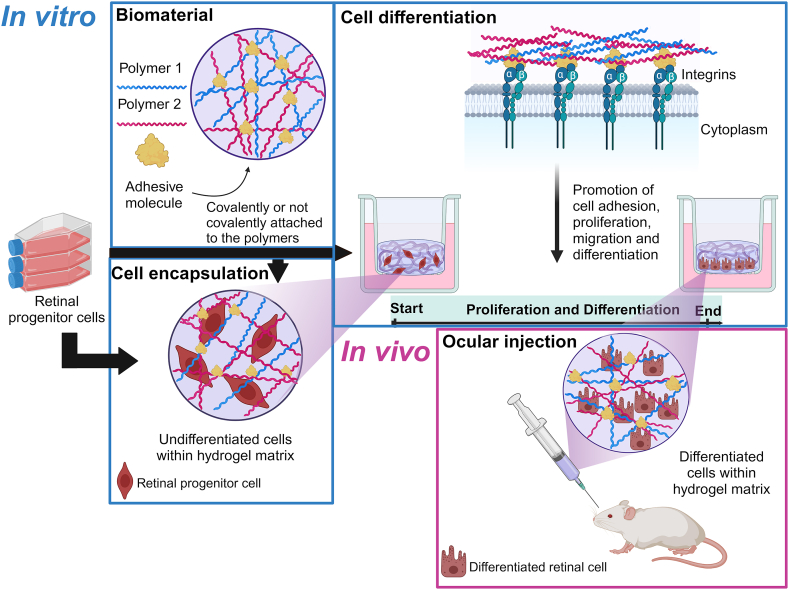
Schematic representation of an exemplary process of cell encapsulation into a hydrogel followed by *in vitro* differentiation and *in vivo* injection. Hydrogel may incorporate adhesive molecules (covalently or non-covalently bound) to enhance cell adhesion. Undifferentiated cells, e.g., RPCs, are embedded within the hydrogel matrix. *In vitro* cell differentiation is stimulated by integrin activation via adhesive molecules, promoting cell adhesion, proliferation, and differentiation. The cell-laden hydrogel is injected intraocularly for the treatment of retinal degenerative diseases. The blue frames correspond to the *in vitro* stages, whereas the pink frames represent the *in vivo* stage. Illustration created with https://BioRender.com. (For interpretation of the references to colour in this figure legend, the reader is referred to the Web version of this article.)

However, in some cases, HA-based hydrogels may require additional biomimetic cues, such as cell-adhesive peptides (Fig. 5), to improve cell attachment, proliferation, and differentiation, suggesting that CD44 receptors alone may not be sufficient [10,11]. For example, Zarembinski et al. covalently attached a maleimide-tagged RGDS peptide to a thiolated HA to form a cell-adhesive hydrogel [22]. The authors demonstrated that the incorporation of the RGDS peptide stimulated the attachment and spreading of ADSCs and BM-MSCs, whereas the scrambled sequence RDGS did not support such cell attachment. In another study, Tang et al. functionalized a GelSH-HAMA hydrogel system with cell adhesive PDA [23]. The introduction of PDA improved cellular adhesion and promoted the differentiation of RPCs into photoreceptors, via activation of the integrin α5β1-PI3K pathway [23].

#### 5.2.2 Cross-linking mechanisms of hydrogels

Injectable cell-laden hydrogels to the eye involve more often chemical crosslinking compared to physical cross-linking. Covalent crosslinking provides hydrogel shape stability post-injection and a longer degradation time. The long-term stability is especially beneficial, as it offers a temporary support while cells start to secrete their own ECM. While this is important for differentiated cells, it becomes even more critical for undifferentiated cells, which typically require an initial *in vitro* culture period to undergo differentiation before being injected *in vivo* (Fig. 5) [23]. The most common reactions are Michael-type addition [[22], [23], [24]] followed by Schiff base reaction [74,33,86] which occur *in situ* without an external trigger. Some authors chose UV-mediated polymerization [75,98], which is not recommended for ocular application as the direct irradiation of UV light may be detrimental for the ocular tissues. In line with this, none of the above-mentioned studies perform *in vivo* testing in orthotopic models. Moreover, UV photopolymerization is a reaction which introduces covalent irreversible bonds, which do not allow injectability after crosslinking. This represents a significant limitation for cell-laden hydrogels which may need *in vitro* preculture to stimulate cell differentiation before *in vivo* injection.

Another approach is based on physical crosslinking mechanisms, like those mediated by the temperature or the presence of ions [21,35,84,87,88,115]. The primary advantage of this approach is the absence of chemical crosslinkers, which may remain unreacted and lead to undesired secondary reactions *in vivo*. However, a notable drawback is the lower stability of physically crosslinked hydrogels that rely on the establishment of weaker interactions compared to covalent bonds, such as hydrophobic interactions, ionic bonds, or hydrogen bridges [138].

A promising concept in the hydrogel field is that of interpenetrating network (IPN) [139]. IPN is defined as a combination of two independent polymeric networks which are physically entangles within each other, without any covalent bond linking them. IPN are usually obtained by forming a polymeric network in the presence of the other, either simultaneously or sequentially. The synergistic combination of polymer properties leads to hybrid systems with improved mechanical properties and swelling behaviour compared to single-network hydrogels [140,141]. For example, Dromel et al.*,* encapsulated RGCs in an IPN hydrogel based on gelatin-hydroxyphenyl propionic acid (GeL-HPA) and tyramine-functionalized hyaluronic acid (HA-Tyr) and crosslinked in the presence of horseradish peroxidase (HRP) and hydrogen peroxide (H_2_O_2_) [89]. Through this hybrid network, they harness the benefits of both polymers, as HA imparts rigidity, stability, and surgical tunability, while GeL enhances cellular adhesion and fosters cell viability [89].

#### 5.2.3 Physical properties of hydrogels

One of the main challenges when using injectable hydrogels for ocular delivery is to control the gelation time and the strength of the hydrogel. For most of chemically crosslinked hydrogels, gelation time and their stiffness follow an opposite trend, as stronger hydrogels tend to gelate more rapidly. Hence achieving hydrogels with desirable properties is not always trivial. This is the case, especially for ocular surface applications, which benefit from quick-gelling yet soft hydrogels [142]. To this aim, Zarembinski et al. introduced oxidized glutathione, a dimer of two disulfide-crosslinked glutathione molecules, into thiol-modified HA and gelatin hydrogels to yield a thiol–disulfide exchange reaction which triggered rapid formation of soft gels [22]. In this system, the oxidized glutathione replaced polyethylene glycol diacrylate (PEGDA) which was previously used as crosslinker with the drawback of causing high hydrogel stiffness for fast-gelling hydrogels [22].

Importantly, gelation time and stiffness are not only interrelated but also connected to cell viability. Encapsulated cells must survive post-injection, endure mechanical stress and replace damaged tissue with healthy and functional new tissue. To this aim, cells need to maintain a healthy metabolic activity and produce their own ECM [74,143]. In this context, the hydrogel must replicate the stiffness of the targeted tissue to provide an adequate biomechanical environment. For example, the stiffness of the retina ranges between 300 Pa and 800 Pa at 37° in saline [144]. The replication of this mechanical parameter in hydrogels designed for retinal regeneration is important to ensure cell viability, preventing exposure to excessive rigidity that could lead to cell death. In line with this, the majority of hydrogels for retinal applications exhibit a stiffness ranging from 200 Pa to 800 Pa, closely approximating the native retinal environment [74,22,23,34,98]. While these values are promising, a subset of studies have employed hydrogels with significantly higher stiffness, around 1 kPa [33,75,89]. Unfortunately, nearly 50 % of the selected studies do not test the hydrogel stiffness, which represents an important limitation.

Hydrogel pore size is a critical parameter influencing cell behaviour and drug delivery within the matrix. The optimal pore size should balance the need to retain cells and bioactive molecules while permitting exchange of nutrients and cellular migration. Although hydrogel degradation can dynamically alter pore dimensions over time, the initial pore architecture significantly impacts the early stages of cell encapsulation and active factors release profiles. Excessive pore size can lead to premature release of cells and encapsulated factors, while overly restrictive pores may reduce cell viability and functionality. Therefore, meticulous pore size engineering is essential for achieving controlled release kinetics and promoting desired cellular outcomes [33,145,146]. Besides this, pore size affects the efficiency of the supply of nutrients within the ECM and the removal of cellular metabolism by-products [75]. This consideration is essential to optimize the microenvironment within the hydrogel, enabling effective metabolic processes and supporting cellular functions [147]. Our examination of the literature identified hydrogels with pore sizes ranging from 35 μm to 180 μm. While none of these studies definitively established an ideal pore size for encapsulating specific cell types, such as RPCs, CjSCs, and CAR-T, it was demonstrated that this range supports nutrient transport and cellular metabolism [74,33,98].

The release of cells from the hydrogel, post-injection, can occur through different mechanisms. A common process entails the degradation of the biopolymers by enzymes normally present in the ECM, such as collagenases and hyaluronidase. Hence, hydrogels made of HA, GeL, or CoL would align with this specific cellular release mechanism [23,34,89]. In some cases, a cell-mediated degradation mechanism may be additionally involved. For example, Dromel et al. developed a hydrogel made of HA and GeL and loaded with RGCs. The degradation of this hydrogel occurs not only through the enzymatic activity of hyaluronidase and collagenases present in the ECM of the retina but also by means of metalloproteinases secreted by RGCs [89]. While several studies have investigated hydrogel degradation *in vitro* [23,34,89], comprehensive *in vivo* degradation assays remain scarce [74,23]. For retinal degenerative diseases, adequate *in vivo* hydrogel stability typically spans from 3 to 4 weeks [74,23]. This duration usually allows ample time for cells to migrate, integrate into the retina, secrete their own ECM, and undergo differentiation, particularly in the case of undifferentiated cells. For example, Jiang et al. conducted subretinal injections of RPCs-laden chitosan/dextran-based hydrogel in mice, and by day 28, they observed RPC cell proliferation, differentiation toward neuron linage (particularly photoreceptors), integration, and tissue regeneration [74]. In the context of anterior segment diseases, the tendency for hydrogel stability is adjusted to approximately 2 weeks [22,88]. Both Zarembinski et al. and Chien et al. pursued a similar approach to address damaged corneas. They conducted subconjunctival injections of ADSCs or iPSCs encapsulated within injectable hydrogels [22,88] that persisted for approximately two weeks within the cornea, demonstrating the reconstruction of injured corneas in both cases. Furthermore, Chien et al. demonstrated that keratocyte-reprogrammed iPSCs encapsulated in CHC hydrogels showed epithelial cell growth in damaged corneas. This approach restored native corneal epithelium thickness and established an endogenous stem cell niche to support long-term corneal epithelial self-renewal [88].

## 6 Injectable hydrogels loaded with nanoparticles

### 6.1 Nanoparticles

Current first-line treatments for posterior segment eye diseases involve frequent IVT injections of FDA-approved anti-VEGF agents [148]. However, these injections face several limitations, including suboptimal patient compliance [149], a high frequency of injections required by the short half-lives of antiangiogenic proteins in the vitreous humor (ranging from 9.82 days for bevacizumab (BVZ) [150] to 5–6 days for AFL [148]), and potential adverse effects, such as cataract formation, endophthalmitis, and rare cases of retinal toxicity and detachment [132]. Therefore, researchers are actively exploring alternative delivery methods for anti-VEGF agents to enhance patient outcomes and reduce the treatment burden [19,20,91,93,96]. NPs offer several advantages over traditional methods, including the opportunity to entrap hydrophilic and hydrophobic drugs [151], the feasibility of being engineered to specifically interact with retinal cells [152], and the protection of sensitive molecules from degradation [153,154]. Moreover, by controlling the size of NPs, the release kinetics and tissue distribution of entrapped molecules can be tuned. Despite their advantageous properties, NPs face challenges in the vitreous. They can interact with macromolecules such as HA, leading to their retention at the injection site or, depending on their size, they can undergo aggregation or rapid clearance from the vitreous. This limits their long-term efficacy and forces multiple injections [[155], [156], [157]]. Additionally, direct injection of NPs can cause toxicity and it is usually associated with a remarkable initial burst [19,20,91,93,95,97,101]. To overcome these limitations, hydrogels loaded with nanoparticles, i.e. nanocomposites, have emerged as a promising delivery platform. By encapsulating NPs within hydrogels, composite systems can be formulated where the hydrogel protects NPs from clearance or aggregation and reduces the burst release of the drug by acting as an additional diffusion barrier.

#### 6.1.1 Encapsulated drugs

Significant research efforts have focused on encapsulating therapeutic agents like BVZ, ranibizumab, sunitinib (SUN), AFL, and p11 anti-angiogenic peptide within MPs- and NPs-loaded hydrogels to address neovascularization in posterior segment eye pathologies [20,92,93,96,97]. The antibody BVZ selectively inhibits the VEGF receptor 2 (VEGFR-2), a key player in neovascularization [93,158]. Ranibizumab is a humanize recombinant monoclonal antibody fragment which specifically inhibits the interaction of VEGF-A with its receptors [159,160]. SUN, a multikinase inhibitor, boasts a broader spectrum of VEGF inhibitory activities, encompassing VEGF receptors 1, 2, and 3 [96]. AFL, a fusion protein, features domains that bind to both VEGFR-1 and VEGFR-2, effectively blocking VEGF signaling [20,92,93,158]. Peptide 11, a novel therapeutic agent, inhibits neovascularization by blocking αVβ3 integrin without eliciting adverse effects [97,161].

**Fig. 6:**
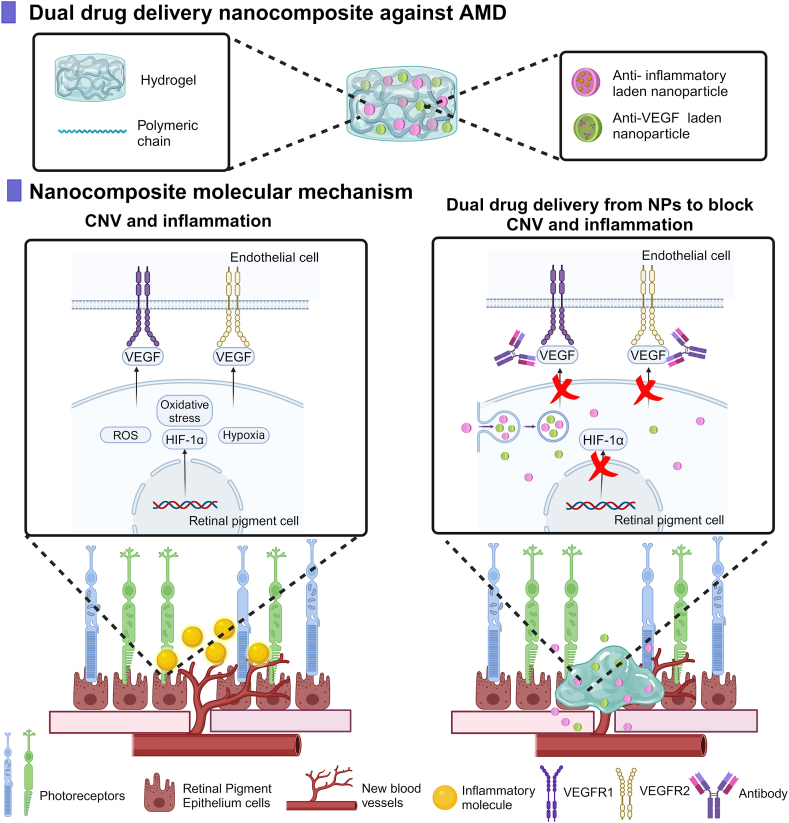
Schematic representation of an exemplary injectable nanocomposite designed to treat AMD using a dual drug delivery strategy. The nanocomposite comprises a hydrogel matrix containing NPs loaded with an anti-inflammatory drug, as well as NPs loaded with an anti-VEGF agent. Upon injection, the NPs are released from the hydrogel and target specific pathways involved in AMD. The anti-inflammatory drug inhibits the HIF-1α pathway to reduce inflammation and the anti-VEGF agent blocks VEGF-induced neovascularization. As an example of drug release, the figure illustrates the internalization of NPs via endocytosis, the most common path. However, NPs may also follow alternative internalization routes or remain outside the cells without being internalized [96,165,166]. Illustration created with https://BioRender.com.

Certain posterior segment eye pathologies feature inflammation in addition to neovascularization. Hence, as graphically represented in Fig. 6, some studies employed a dual therapeutic approach, combining an anti-VEGF factor with an anti-inflammatory drug like dexamethasone (Dexa) or a hypoxia-inducible factor (HIF-1) inhibitor [20]. For example, AFL and Dexa loaded into poly-lactide-*co*-gycolic acid (PLGA) microparticles (MPs) and NPs, respectively, and, subsequently embedded into a (PEG-PLLA-DA)-(NIPAAm) thermoresponsive hydrogel, provided a sustained release of AFL and Dexa for over 224 days [20]. In another study, liposomes loaded with SUN and acriflavine (ACF), an HIF-1α inhibitor, which plays a crucial role in reducing the expression levels of vasoactive agents, and incorporated into a HA-based hydrogel, allowed a 70 % release of SUN in 15 days and a 77 % release of ACF in the same period of time [96]. In a different study, BVZ was loaded into Ch NPs, whereas Dexa was directly included within the hydrogel [93]. As a result, Dexa was found to be completely released from the hydrogel within 20 h *in vitro*, while BVZ required 10 days to reach a complete release. This combined formulation synergistically enhanced the efficacy of BVZ in slowing down the progression of neovascularization in a rat model [93]. In other cases, NPs themselves can reduce inflammation by scavenging ROS. For example, PDA NPs are being explored to reduce inflammation in various ocular diseases affecting both the outer retina (such as AMD and DR) and the inner retina (like glaucoma) [162]. The abundance of phenolic, catecholic and quinonoid groups in PDA provides its ROS scavenging capabilities, allowing it to mitigate ROS-mediated injury and inflammation [163,164]. Liu et al. developed an injectable photocrosslinkable hydrogel based on modified GeL containing PDA NPs. Their *in vitro* studies on a retinal ganglion cell line and *in vivo* studies in a mouse model demonstrated the ROS scavenging capability and consequent anti-inflammatory effects of these NPs [85].

The encapsulation of small molecules is generally applied to other types of eye diseases, like uveal melanoma and autoimmune uveitis, where neovascularization is not the main feature of the pathology [90,91,95]. In these cases, the strategy addresses inflammation, abnormal cell growth, or immune system responses. For example, Zhu et al. showed that minocycline, a compound with antibiotic and anti-inflammatory properties, can co-assemble with a phosphorylated peptide in the presence of Ca^2+^ [90].This nanocomplex was loaded into a thermosensitive hydrogel, providing sustained release for three weeks *in vitro* and a significant reduction in ocular inflammation *in vivo* [90].

#### 6.1.2 Factors influencing the biodistribution of nanocarriers and the access of drugs to their targets

PLGA NPs stand out as the most commonly used particles for the formulation of nanocomposite hydrogels for ocular delivery [20,92,94,95,97,101] followed by liposomes [91,96], chitosan NPs [93], which are well-studied carriers in ocular applications [167,168], and peptide NPs [90] (Table 2). The interest for PLGA particles is due to their biodegradability, and possibility to tune degradation and release properties [[169], [170], [171]]. Nevertheless, protein denaturation can occur during the encapsulation and also during the release process [97].

**Table 2:** Hydrogels loaded with drug-laden NPs. Description of the hydrogel building blocks, crosslinking mechanism (“*Chem*” and “*Phys*” indicate chemical and physical crosslinking, respectively) nano- and microparticle type (“NPs” and “MPs”, respectively), encapsulated factor, targeted disease and animal model.

Hydrogel building blocks	Crosslinking mechanism	Nano- (NPs) or microparticle (MPs)	Encapsulated factor	Disease and Animal model	Ref.
PEG-PCL-PEG	Thermogelation *(Phys)*	PLGA NPs	P11 peptide in PLGA Nps	Retinal degenerative diseases –	Toit et al., 2021 [97]
PEG-PLLA-DA-NIPAAm	Thermogelation *(Phys)*	PLGA MPs PLGA NPs	AFL in PLGA MPs Dexa in PLGA NPs	Age-related macular degeneration –	Rudeen et al., 2022 [20]
pNIPAAm-PEGDA	Thermogelation (*Phys*)	PLGA MPs	AFL or ranibizumab in PLGA MPs	Retinal degenerative diseases –	Osswald et al., 2017 [92]
HA-PF407	Thermogelation *(Phys)*	HA-coated PLGA-DOTAP NPs	–	Retinal degenerative diseases Mouse model	Ottoneli et al., 2022 [94]
CoL-HA-PEG	Thermogelation *(Phys)*	mPEG-PLGA NPs	Curcumin in mPEG-PLGA NPs	Uveal melanoma –	Xie et al., 2021 [95]
mPEG-PCL-PLA-PCL-mPEG	Thermogelation *(Phys)*	PCL-PLA-PEG-PLA-PCL NPs	IgG-Fab in PCL-PLA-PEG-PLA-PCL NPs	Retinal degenerative diseases –	Agrahari et al., 2016 [19]
PF127-HPMC	Thermogelation *(Phys)*	CNAC NPs	BVZ in CNAC NPs. Dexa in PF127-HPMC hydrogel	Diabetic retinopathy Rat model	SL Taheri et al., 2022 [93]
PDLLA-PEG-PDLLA	Thermogelation *(Phys)*	Mino/NapGFFpYNPs	Minocycline in Mino/NapGFFpY NPs	Autoimmune uveitis Rat model	Zhu et al., 2022 [90]
Dopamine-Alg-Ca^+2^	Ionic gelation *(Phys)*	HA NPs	LAP and DOX co-loaded in NPs	Uveal melanoma Mouse model	Guo et al., 2024 [172]
NapFFKK	Supramolecular bonding *(Phys)*	Micelles	Rapamycin in micelles Levofloxacin hydrochloride in hydrogel	Corneal graft rejection Rabbit model	Xu et al., 2024 [173]
HAVS-HASH	Thiol-ene chemistry *(Chem)*	PLGA NPs	BSA in PLGA NPs	Age-related macular degeneration –	Hsu et al., 2021 [101]
HAvinyl-p(CBMA-co-AC)SH	Thiol-ene chemistry *(Chem)*	Liposomes	SUN and ACF co-loaded in liposomes	Retinal degenerative diseases Rat model	Li et al., 2022 [96]
HAADH-PEGDE-HAMA	1. Alkylation *(Chem)* 2.UV -polymerization (*Chem)*	Liposomes	Latanoprost in liposomes	Glaucoma –	Widjaj et al., 2013 [91]
Gel-CA	UV-polymerization *(Chem)*	PDA NPs	Curcumin in PDA NPs	Oxidative retinal damage Mouse model	Liu et al., 2024 [85]
GeL-oxDex-borax	Schiff-Base Borate-diol ester bonding *(Chem)*	PEG-PLGA NPs	Probucol	Corneal wounds Rat model	Ge et al., 2024 [174]
ACD-NHA	Schiff base *(Chem)*	HA-coated Ch NPs	Dexa in NPs and GCV in ACD-NHA hydrogel	Acute retinal necrosis –	Zhang et al., 2024 [175]

ACD, aldehyde β-cyclodextrin; ACF, acriflavine; AFL, aflibercept; Alg, alginate; BVZ, bevacizumab; CNAC, chitosan-N-acetyl-L-cysteine; CoL, collagen; DA, diacrylate; Dexa; dexamethasone; DOTAP, (1,2-Di-(9Z-octadecenoyl)-3-trimethylammonium propane methylsulfate; DOX, doxorubicin; GCV, ganciclovir; GeL, gellatin; Gel-CA, cinnamic acid-conjugated gelatin; HA, hyaluronic acid; HAADH, hydrazide hyaluronic acid, HAC; hyaluronic acid methylcellulose; HPMC, hydroxypropyl methylcellulose; HASH, thiolated hyaluronic acid; HAvinyl, vinyl hyaluronic acid; HAVS, divinyl sulfone-functionalized hyaluronic acid; IgG-Fab, immunoglobulin G fragment antigen binding; LAP, lapatinib; mPEG, methoxy-poly(ethylene-glycol); NapFFKK: peptide (Nap = Naphthalene group, F = Phenylalanine, F = Phenylalanine, K = Lysine, K = Lysine); NapGFFpY, phosphorylated peptide; p(CBMA-co-AC)SH; thiolated copolymer of carboxybetaine methacrylate; NHA = aminated HA; PCL, polycaprolactone; PDA, polydopamine; PDLLA, poly(D,L-lactide); PEG, polyethylene glycol; PEGDE, poly(ethylene glycol) diglycidyl ether; PLA, polyglycolic acid; PLGA, poly-lactide-co-glycolic acid; pNIPAAm; poly(N-isopropylacrylamide); SUN, sunitinib.

The character “-” in the animal model column indicates that no animal model has been reported.

As an alternative, a few studies propose the use of liposomes, which can accommodate both hydrophilic and hydrophobic drugs [167], in nanocomposite injectable hydrogels for ocular delivery [91,96]. This was exploited, by Li et al., for the co-delivery of the hydrophilic SUN and the hydrophobic ACF [96]. By embedding liposomes into a hydrogel, a >70 % release of SUN and ACF over 15 days was reached. Similarly, in another study, a formulation of liposomes containing latanoprost enabled a sustained release over a period of three weeks [91].

One of the primary concerns regarding the biodistribution and toxicity of NPs is their interaction with proteins [176]. Protein adsorption on the NP surface (protein corona formation) could destabilize the NPs leading to aggregation. Aggregated NPs may fail to reach their target tissues and display reduced bioavailability. Moreover, aggregation can also obstruct microcirculation in the eye, and can cause accumulation in non-target tissues, potentially leading to toxicity and inflammation [177]. In the case of the ocular surface, NP aggregation may also hinder lacrimal drainage [178].

IVT injection has emerged as the preferred administration route for nanocomposite hydrogels. IVT injection delivers the formulation to the vitreous humor, which makes it adequate when the targeted tissue is the inner retina, such as in glaucoma (Fig. 4). However, when the outer retina is targeted, the efficacy of the treatment depends on the ability of the NPs or the released factors to migrate from the vitreous to the outer retina. The success of this process may depend on clearance mechanisms, pathological microenvironment, type of particles and drugs.

To increase the delivery to the outer retina, the IVT injection may be replaced by the subretinal injection, which directly positions the formulation in the subretinal space. However, subretinal injection is technically more demanding, increasing the risk of side effects associated with the injection procedure. The trade-off between delivery efficiency and invasiveness may explain why IVT injection remains the preferred approach for hydrogel-based nanoparticle therapies.

Another possible administration route is the sub-tenon's injection, a periocular approach involving the direct injection of the formulation into the region outside the sclera. This route bypasses the vitreous humor, which may interfere in the drug delivery to the target tissue. For example, Li et al. investigated the effectiveness of a sub-tenon's injection in delivering an anti-VEGF agent and a HIF-1α inhibitor co-loaded in liposomes within a HA-based hydrogel [96]. They observed a reduction in the retina's thickness in a CNV rat model 28 days after the injection, and a reduced expression of four neovascularization markers, i.e., AKT, mTOR, HIF-1α, and VEGF [96].

NPs size is another critical parameter influencing the efficacy in reaching the target tissue. Particle size can be strategically modulated to control particle fate after ocular injection. Preliminary studies suggest that particles larger than 500 nm tend to be rapidly cleared from the vitreous to the aqueous humor flow, whereas smaller NPs, better if in the 200–250 nm range, exhibit slower clearance being able to penetrate the inner retina and offering an enhanced delivery potential [179,180]. Most of the studies reported the use of NPs with an average diameter between 100 and 350 nm [19,20,91,93,95,97,101], and only two studies employed MPs to deliver an anti-VEGF agent *in vitro* [20,92]. The choice on particle size may not only affect NP clearance, but also the extent of NP penetration through the retina. For example, a study by Remaut et al. utilizing a novel *ex vivo* bovine explant model that maintained the vitreous-retinal attachment, determined that retinal penetration is size-dependent. Testing polystyrene beads of 40 nm, 100 nm, and 200 nm, the researchers found that only the 40-nm beads were capable of crossing the vitreoretinal interface and achieving significant retinal penetration, with over 30 NPs per 1000 μm^2^ of the retina [181].

Although the biodistribution of NPs is critical for understanding their performance, unfortunately only two studies addressed this aspect, with the limitation that they simply rely on fluorescence images. For example, using fluorescence through tissue scanning microscopy (TSM) and confocal laser scanning microscopy (CLSM), Li et al. studied the biodistribution of a hydrogel containing liposomes, after sub-tenon's injections in rats [96]. Their findings revealed that free drugs had limited penetration through the sclera and choroid, resulting in low bioavailability. In contrast, an improved drug delivery to the retinal layers and choroid, was attributed to the retention of the hydrogel and to the increased liposome penetration [96]. Ottonelli et al. conducted a study to compare the *in vivo* biodistribution of HA-coated and uncoated NPs loaded in a hydrogel [94]. Using CLSM and fluorescently labelled NPs, they observed distinct behaviours for the two given formulations. Uncoated NPs, which were positively charged, were primarily retained in the vitreous due to interactions with the anionic biomacromolecules present there. In contrast, HA-coated NPs, with their negative charge, were found to penetrate the retinal layers. This suggests that the HA coating reduced the interaction between the NPs and the vitreous macromolecules, allowing for improved retinal delivery [94]. These findings are in line with the quantitative analysis performed by Remaut et al., who employed single-particle tracking microscopy (SPT) to quantitatively assess nanocarrier mobility in an *ex vivo* bovine vitreous model following IVT injection [182]. They observed that positively charged NPs exhibited a heterogeneous diffusion profile, with a significant portion becoming immobilized within CoL fibrils. Hydrophobic NPs demonstrated reduced mobility. Conversely, negatively charged surfaces increased the mobile fraction of NPs within the vitreous [182]. In conclusion, alongside nanoparticle size, physicochemical characteristics play a crucial role in their mobilization through the vitreous and subsequent retinal penetration.

The NP shape is also a critical parameter [183]. Studies have revealed that shape dictates NPs cellular internalization, biodistribution, and interaction with immune cells. Specifically, non-spherical nanoparticles tend to be less readily taken up by cells than spherical ones [184].

Nanoparticle clearance and penetration into the retina are further affected by particle interaction with two anatomical structures, *i.e.*, the inner limiting membrane and the blood-retinal barrier. The inner limiting membrane is mainly formed by the basement membrane of the Müller glial cells, and acts as a boundary separating the vitreous from the retina. Being a dense and organized membrane, it may restrict the penetration of certain components toward the retina [185]. The BRB consists of tight junctions between retinal capillary endothelial cells (inner BRB) or RPE cells (outer BRB) [186,187]. BRB may regulate the clearance of intravitreally injected components, by directing them from the retina to the choroidal circulation, hence reducing their residence time in the retina [184,186,188]. Finally, although the eye is considered an immune-privileged site, it still contains resident immune cells, which through phagocytosis, may direct intravitreally injected NPs to regional lymph nodes leading to a lymphatic-like drainage [189]. This may cause reduced intravitreal retention of nanoparticles, increase their systemic exposure and possible immunogenicity [177].

**Fig. 7:**
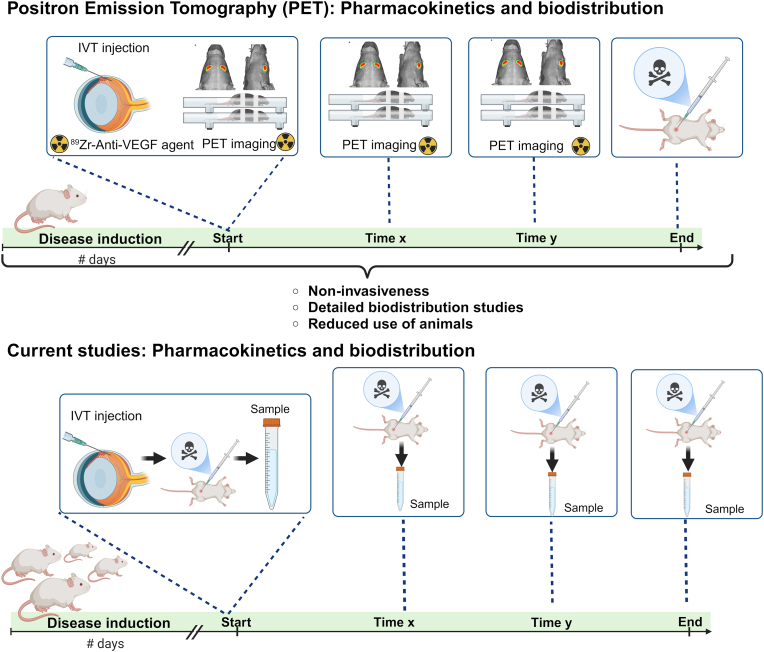
Comparison of PET imaging and conventional pharmacokinetic studies in ocular drug delivery. PET has emerged as a non-invasive method for studying drug pharmacokinetics in the eye, reducing animal use, and providing detailed biodistribution data. Conventional methods rely on post-mortem tissue sampling, requiring more animals. Illustration created with https://BioRender.com.

Conventional methods for studying IVT pharmacokinetics rely on sacrificing animals at defined time points to collect vitreous humor samples (Fig. 7) [190,191]. This approach restricts the number of data points and raises ethical concerns. Therefore, Positron Emission Tomography (PET) has emerged as a powerful non-invasive tool for evaluating drug biodistribution within the eye, reducing the number of animals used (Fig. 7) [192,193]. Radiolabelling of antibodies has been extensively exploited in oncology (ImunoPET) [194,195]. However, only a few studies employed this technique in the ocular field. For example, Luaces-Rodríguez et al. studied the ocular and blood pharmacokinetics of IVT injections of BVZ and AFL, both radiolabelled with zirconium-89 (^89^Zr) in rats. They found that both antibodies remain in the ocular cavity for up to 12 days [196]. Another example was the radiolabelling of adalimumab, an anti-TNFα drug, with ^89^Zr. The pharmacokinetic profile and the biodistribution was measured by PET imaging in a uveitis rat model. This study found that the ^89^Zr-adalimumab remains around two times longer in rats with the disease compared to healthy ones [197]. The reason behind this phenomenon, is the overproduction of Tumor Necrosis Alpha (TNF-α) by macrophages, which infiltrate to the blood-retina barrier during uveitis [198]. The high selectivity and affinity of adalimumab to TNF-α reduced two times their clearance from the eyes [197]. As a matter of fact, the use of PET in the ocular field is currently limited to the injection of free radiolabelled drugs. To the best of our knowledge, no studies so far reported the use of PET for drug-loaded nanoparticles or drug-loaded nanocomposites. In our opinion, PET may offer a great tool for the non-invasive evaluation of the biodistribution of drugs delivered through nanocomposites in the future landscape of the field.

### 6.2 Nanocomposite hydrogels for intra-ocular injection

#### 6.2.1 Building blocks and cross-linking

In contrast to cell-laden hydrogels (section 5), for which mimicry of the ECM was sought by using preferably natural polymers, nanocomposite hydrogels often rely on synthetic building blocks which allow a more precise control over material properties, such as injectability, stiffness, porosity, degradation time, among others. Examples of synthetic polymers include block copolymers mainly used for their thermo-sensitive behavior, such as methoxy-poly-(ethylene glycol)−poly(caprolactone)−poly(lactic acid)−poly-(caprolactone)−poly(ethylene glycol)-methoxy (mPEG-PCL-PLA-PCL-mPEG) [19], polyethylene glycol-polycaprolactone-polyethylene glycol (PEG-PCL-PEG) [97], poly(ethylene glycol)-poly(L-lactic acid) diacrylate (PEG-PLLA-DA)-*N*-isopropylacrylamide (NIPAAm) [20], poly(D,L-lactic acid)-poly(ethylene glycol)-poly(D,L-lactic acid) (PDLLA-PEG-PDLLA) [90] and poly(ethylene oxide)-poly(propylene oxide)- poly(ethylene oxide) (PEO-PPO-PEO) [93,94]. When including natural building blocks, hydrogels are generally made of polysaccharides, like HA, or more rarely of proteins like Col, used in their native form or chemically modified to introduce reactive groups allowing gelation [90,95,101]. In hybrid hydrogels, made of a mixture of synthetic and natural polymers, the synthetic component is often a thermosensitive block copolymer allowing thermogelation [93,94].

For nanocomposite hydrogels, physical gelation stands out as the most common cross-linking mechanism (Table 2). Notably, almost all physical hydrogels reported are thermosensitive, hence they can undergo gelation upon exceeding their polymer low critical solution temperature (LCST). This threshold usually lies close to the physiologic temperature of the human body (37 °C), allowing the hydrogels to remain in solution, hence being injectable at room temperature, and gellify at body temperature after injection. Using these hydrogels in an *in vivo* situation, crosslinking occurs *in situ* upon IVT injection [19,20,92,94,95,97]. An interesting exception is represented by the study of Xu et al. describing a supramolecular hydrogel based on the peptide NapFFKK, where physical gelation, via self-assembly, is obtained by supramolecular bonding, including π-π stacking interactions, hydrophobic interactions, hydrogen bonds, and electrostatic interactions [173]. Only a few articles follow a chemical crosslinking strategy involving click chemistry [96,101] or UV photopolymerization [91]. As highlighted in section 5, the utilization of UV photopolymerized hydrogels for ocular delivery raises concerns, as direct UV irradiation on the eye has the potential to cause tissue damage. On the other side, injectability of pre-crossinked hydrogels may be challenging given the irreversibility of the UV-mediated chemical cross-linking.

## 7 Personalized medicine and challenges in clinical translation

Injectable hydrogels loaded with nano-encapsulated drugs or cells offer a potential platform for personalized medicine in ocular diseases by, *e.g.*, enabling customization of drug release kinetics and dosage, as well as personalized cell administration and control of the extracellular micro-environment, which can be potentially tuned based on the specific patient's condition, the disease progression and the physiopathology of the disease. Firstly, the rate at which a hydrogel degrades and releases drugs or cells can be adjusted modifying the hydrogel cross-linking density [81,84]. For nanoparticle-loaded hydrogels, nanoencapsulation further helps to fine-tune the release. For cell-loaded hydrogels, the adjustment of the crosslinking density can enable a customized hydrogel stiffness and stability, which can be modulated as a function of the cell type/density and disease progression [83]. It is indeed known that cell differentiation is affected by the mechanical stimulation of the surrounding matrix in a cell-type dependent fashion [77]. Secondly, stimuli-sensitive hydrogels can change their properties in response to ocular conditions (*e.g*., tear pH, intraocular temperature or pressure, light, enzymes, or glucose levels) enabling a self-regulated drug release [199]. Thirdly, injectable hydrogels can be pre-loaded with personalized drug concentrations or cell density, ensuring that each patient receives an optimized dosage. Moreover, patients with complex ocular diseases (e.g., diabetic retinopathy, AMD) that require multiple drugs, may benefit from the possibility of loading the hydrogel with different drugs with distinctive release profiles [81,82]. Furthermore, some smart hydrogels can be reloaded with drugs through subsequent injections without requiring surgical replacement (on-demand refill systems) [200]. Finally, several injectable hydrogels can be optimized to be processed by 3D bioprinting to match the patient's unique ocular anatomy for optimal retention and efficacy [80,[201], [202], [203]].

Despite the several promising features of intraocular hydrogels, their clinical translation faces technological challenges in manufacturing and large-scale production, regulatory aspects, and long-term safety. Due to the complexity and variability of hydrogel fabrication across different systems, the estimated development costs through clinical translation range from $50 million to $800 million [200].

A significant obstacle to the clinical translation is ensuring compatibility with current good manufacturing practices (cGMPs). The typical small-batch synthesis of hydrogels at the preclinical stage needs substantial efforts to scale up fabrication and synthesis strategies. This scale-up inevitably introduces challenges related to batch variations, robustness, safety, and efficiency. Natural polymer hydrogels face further complexities due to the inherent heterogeneity of natural polymers, which can lead to variations in molecular-scale properties and potentially affect the final hydrogel characteristics. Furthermore, the high water content of hydrogels complicates sterilization, storage, and fabrication processes [204].

Obtaining FDA and/or European Medicines Agency (EMA) approval for clinical trials and market authorization is an extensive regulatory process requiring robust evidence of safety and efficacy. In contrast, current studies on cell- and nanoparticle-laden hydrogels lack robust *in vivo* testing methods for such complex systems, which makes it difficult to predict their effectiveness and safety in future clinical settings. The ocular delivery of these systems has been mainly tested in mice and rats employing ectopic and orthotopic models to test biocompatibility and efficacy, respectively. Notably, several studies on NPs-laden hydrogels lack *in vivo* studies, highlighting the earlier stage of evaluation for these systems, compared to cell-laden hydrogels. None of the selected studies reported the use of rabbits for the drug/cell delivery to the eye posterior segment. Despite the higher cost, the rabbit model offers several advantages, such as the possibility of better studying age-related eye conditions due to the longer lifespan, easier injections, and more extensive tissue sampling due to larger eye size [205]. Non-human primates are uniquely valuable in vision research, due to possession of macula. However, their use is extremely limited by the need for specialized laboratory facilities, and high costs [206]. This gap in preclinical testing underscores the need for robust *in vivo* data to bridge the translational gap between the bench and clinics.

Focusing on the possibilities toward clinical translation, it is important to clarify that there are no FDA approvals or clinical trials for ocular nanocomposites, which are the systems that have demonstrated the most significant increase in publication trends over the past five years in preclinical research. This reveals a substantial gap between preclinical studies and clinical applications, likely attributed to the complexity of these delivery systems, which involve two distinct drug delivery mechanisms, each influencing the release profile [207]. In contrast, there are a few examples of ocular delivery systems which combine hydrogels with drugs and are FDA-approved or under clinical trials. For example, there is an ongoing phase I clinical trial to evaluate the safety, tolerability, and efficacy of the hydrogel system OTX-TKI for IVT injection, in subjects who suffer from wet-AMD [208]. The OTX-TKI system is a hydrogel implant based on PEG fibers with dispersed microcrystals of Axitinib, a small tyrosine kinase inhibitor (TKI), known for its anti-angiogenic properties. This system provides a prolonged release profile up to 6 months, targeting chronic neovascularization [209]. ReSure® sealant is also a PEG-based hydrogel, approved by the FDA in 2014 that creates a temporary, soft, and lubricious sealant to prevent fluid egress following cataract or intraocular lens placement surgery [210]. It is the only FDA-approved sealant indicated in closing a leaking corneal incision, demonstrating a rate of less than 1 % in corneal wound leakage [211]. Furthermore, in 2018 the FDA approved the use of Dextenza®, an intracanicular hydrogel implant, based on PEG, for the prolonged delivery of Dexa over 1 month, targeting the inflammation after ophthalmologic surgery and allergic conjunctivitis [212]. Generally speaking, these systems have been designed with relatively straightforward compositions and well-defined release mechanisms (when containing drugs), which have facilitated their regulatory approval and clinical adoption. As an example of their relative simplicity, ReSure® does not even contain a drug. In general, their formulations balance biocompatibility, ease of administration, and simple composition, which are essential for clinical translation. In contrast, the new generation of more complex systems described in this review, *i.e.*, hydrogels loaded with one or more types of nano-encapsulated drugs or cells, is still under pre-clinical investigation. These more sophisticated systems offer enhanced tunability and multi-level control over drug/cell release kinetics, targeting, and tissue interaction. However, this added complexity introduces new challenges, including more difficult manufacturing, potential safety concerns, and a more demanding regulatory pathway.

Regarding cell therapy in ophthalmology, there is no clinical trial nor FDA-approved cell-laden injectable hydrogel treatment. However, to pave the way in this direction there are numerous clinical trials focusing on the subretinal transplantation of several cell types, such as Human Embryonic Stem Cell-Derived Retinal Pigmented Epithelial (hESC-RPE) cells, aiming to regenerate the damaged RPE layer in degenerative retinal diseases [[213], [214], [215]]. Most of these studies employ cells dispersed in a saline solution, whereas only one trial utilizes a PLGA patch supporting cell growth. In this study, participants undergo sub-retinal transplantation of autologous induced pluripotent stem cell-derived retinal pigment epithelium (iRPE) [216].

Considering all this information, we believe that future efforts in the design and evaluation of cell-laden hydrogels and NP-laden hydrogels will be crucial to transform the current treatment paradigm in ophthalmology. These innovations have the potential to bring long-acting biodegradable systems for treating a wide range of ocular diseases.

## 8 Conclusions

Ocular diseases are experiencing a global increase in prevalence, due to aging populations and lifestyle changes. A promising approach involves the development of new delivery systems for cells and drugs, designed to avoid surgical interventions while enhancing patient compliance. Injectable hydrogels are a promising remedy to these challenges if choosing the appropriate biomaterial properties. This comprehensive review encompasses a spectrum of injectable hydrogels loaded with cells or drug-loaded NPs to seek ocular tissue regeneration and sustained drug delivery. On one side, hydrogels engineered to mimic the ECM of encapsulated cells facilitate the expression of typical markers and metabolic processes, thereby fostering the restoration of damaged ocular tissue. The integration of adhesive molecules within hydrogels amplifies cell-biomaterial adhesion, cellular interconnections and cellular differentiation. On the other side, the combination of NPs and hydrogels allows sustained release, prevents rapid clearance and enables the inclusion of hydrophobic drugs that cannot be directly incorporated into the hydrophilic hydrogel network. Future studies will have to fill the gap between the preclinical development and the clinical translation of these sophisticated systems. This will demonstrate the feasibility or the overcomplication of these strategies toward the clinical application.

## CRediT authorship contribution statement

**Elena Ibeas Moreno:** Writing – original draft, Visualization, Investigation. **María José Alonso:** Writing – review & editing, Methodology, Funding acquisition, Conceptualization. **Anna Abbadessa:** Writing – review & editing, Visualization, Supervision, Project administration, Methodology, Funding acquisition, Conceptualization.

## Declaration of generative AI and AI-assisted technologies in the writing process

During the preparation of this work the authors used ChatGPT by OpenAI solely to enhance the readability and improve the language clarity of some parts of the manuscript, without altering the original content or intent. After using this tool, the authors reviewed and edited the text as needed and take full responsibility for the content of the published article.

## Declaration of competing interest

The authors declare the following financial interests/personal relationships which may be considered as potential competing interests: Maria Jose Alonso reports a relationship with Pharmamix Vision that includes: funding grants. Other authors declare that they have no known competing financial interests or personal relationships that could have appeared to influence the work reported in this paper.

## Data Availability

Data will be made available on request.

## Abbreviations

ACF: Acriflavine
ADSCs: Adipose-derived stem cells
AFL: Aflibercept
AKT: Protein kinase B
Alg: Alginate
AMD: Age-Related Macular Degeneration
AP: Alginate-poly-L-lysine
ARPE-19: A retinal pigment epithelium cell line
BM: Bone Marrow
BRB: Blood Retinal Barrier
BSA: Bovine serum albumin
BVZ: Bevacizumab
CAC: Composite alginate collagen
CAR-T: Chimeric antigen receptor T
Ch: Chitosan
CHC: Carboxymethyl-hexanoyl chitosan
CoL: Collagen
CHC: Carboxymethyl-hexanoyl chitosan
CjSCs: Conjunctival stem cells
CNAC: Chitosan-N-acetyl-L-cysteine
CNV: Choroidal neovascularization
CS: Chitosan hydrochloride
DA: Diacrylate
Dex: Dextran
Dexa: Dexamethasone
DOTAP: (1,2-Di-(9Z-octadecenoyl)-3-trimethylammonium propane methylsulfate
DR: Diabetic retinopathy
ECM: Extracellular matrix
ERK: Extracellular signal-regulated kinase
ESCs: Embryonic stem cells
FDA: Food and Drug Administration
GCMSs: Gelatin methacrylic chitosan microspheres
GDNF: Glial cell-derived neurotrophic factor
GeL: Gelatin
GeLMa: Gelatin methacrylamide
GeLSH: Thiolated gelatin
GG: Gellan gum
HA: Hyaluronic acid
HAMA: Methacrylated hyaluronic acid
HAMC: Hyaluronic acid methylcellulose
HASH: Thiolated hyaluronic acid
HAVS: Divinyl sulfone hyaluronic acid
HEK293: Human embryonic kidney 293 cell line
hESCs: Human embryonic stem cells
hESC-RPE: Human Embryonic Stem Cell-Derived Retinal Pigmented Epithelial
HIF-1: Hypoxia-inducible factor-1
H2O2: Hydrogen peroxidase
HPA: Hydroxyphenyl propionic acid
HPMC: Hydroxypropyl methylcellulose
HRP: Horseradish peroxidase
hRGCs: Human retinal ganglion cells
IgG-Fab: Immunoglobulin G fragment antigen binding
IOP: Intraocular pressure
iPSCs: Induced pluripotent stem cells
IPN: Interpenetrating network
iRPE: Induced pluripotent stem cell-derived retinal pigment epithelium
IVT: Intravitreal
LCST: Lower critical solution temperature
LSCs: Limbal stem cells
MC: Methylcellulose
MAPK: Mitogen-activated protein kinase
mPEG: Methoxy-poly(ethylene glycol)
MSCs: Mesenchymal stem cells
NPs: Nanoparticles
oxDex: Oxidized dextran
oxPEG: Oxidized polyethylene glycol
p(CBMA-co-AC)SH: thiolated copolymer of carboxybetaine methacrylate
PCL: Polycaprolactone
PDA: Polydopamine
PEG: Polyethylene glycol
PEGDA: Polyethylene glycol diacrylate
PEGDE: Polyethylene glycol diglycidyl ether
PI3K: Phosphatidylinositol-3-kinase
PLA: Polylactic acid
PLGA: Poly-lactide-co-gycolic acid
PLLA: Poly-L-lactic acid
Pkc-α: Protein kinase C- α
pNIPAAm: Poly(N-isopropylacrylamide)
RGD: Arginine-glycine-aspartate
ROS: Reactive oxygen species
RP: Retinitis pigmentosa
RPCs: Retinal Progenitor Cells
RPE: Retinal Pigment Epithelium
RSC: Retinal Stem cell
SUN: Sunitinib
SVEGFR1: Secrete vascular endothelial growth receptor 1
TKI: Tyrosine kinase inhibitor
TNF-α: Tumor Necrosis Factor α
Tyr: Tyramine
UV: Ultraviolet
VEGF: Vascular endothelial growth factors
WHO: Word Health Organization
89 Zr: Zirconium 89
4SPEG: Poly(ethylene glycol) ether tetrasuccinimidyl glutarate
